# Suprapterional keyhole approach for anteromedial skull base lesions: How I do it

**DOI:** 10.1007/s00701-024-06202-y

**Published:** 2024-07-31

**Authors:** Toshiaki Inomo, Kenichiro Iwami, Tadashi Watanabe, Koji Osuka

**Affiliations:** 1 Department of Neurosurgery, Aichi Medical University, Aichi, Japan; 2 Department of Neurosurgery, Nagoya University Graduate School of Medicine, 65 Tsurumai-cho, Showa-ku, Nagoya, 466-8550 Japan

**Keywords:** Suprapterional keyhole, Exoscope, Endoscope, Anterior cranial fossa, Planum sphenoidale, Olfactory groove

## Abstract

**Background:**

For a minimally invasive treatment approach to the anteromedial part of the anterior cranial fossa (ACF), a small incision and craniotomy of the posterolateral part of the ACF are preferable.

**Method:**

We described the concept and technique of suprapterional keyhole approach (SPKA), which uses an exoscope and endoscope to treat ACF lesions.

**Conclusion:**

The SPKA enables ACF observation from the lateral direction; the endoscope’s extended viewing angles enable the observation of the anteromedial part of the ACF, including the bilateral olfactory groove. Facial skin and large scalp incisions are avoided, making this approach efficient for ACF lesions.

**Supplementary information:**

The online version contains supplementary material available at 10.1007/s00701-024-06202-y.

## Relevant surgical anatomy

**Fig. 1:**
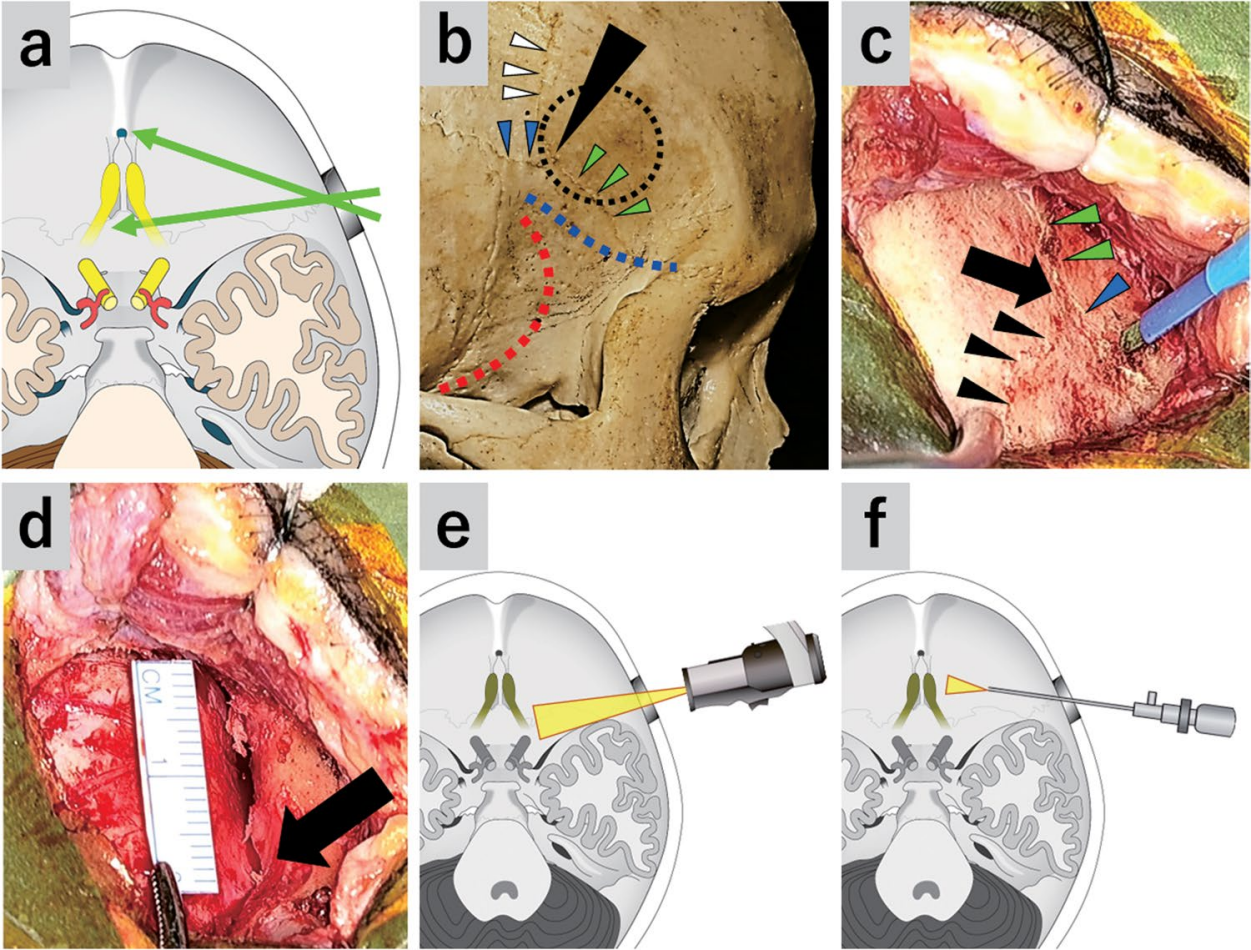
Illustrations and photographs of the SPKA. **a** Surgical corridor of the SPKA. **b** A dry skull. Black arrow head, ICP; white arrow heads, coronal suture; blue arrow heads, sphenoparietal suture; green arrow heads, sphenofrontal suture; blue dotted line, ACF; red dotted line, middle cranial fossa; black dotted circle, a keyhole craniotomy at the posteroinferior edge of the ACF. **c** A small scalp incision. Black arrow, ICP; black arrow heads, coronal suture; green arrow heads, sphenofrontal suture; blue arrow head, sphenoparietal suture. **d** 2 cm craniotomy. Black arrow, ICP. **e** The use of a 3D exoscope to observe sphenoid planum. **f** The use of an endoscope to observe the olfactory groove or more anterior part that are difficult to observe with an exoscope

To work on the anteromedial ACF without facially skin incisions or opening the frontal sinuses, a small scalp incision and craniotomy at ACF’s posterolateral part are preferable (Fig. 1a). The frontal lobe’s inferolateral surface comprises the inferior frontal gyrus, while the coronal suture runs over the inferior frontal gyrus based on previous craniometric reports [1, 6]. Moreover, the inferior coronal point (ICP) coincides with the position of the trigone of the inferior frontal gyrus (Fig. 1b) [6, 2].

## Description of the technique

Before the operation, the facial nerve’s temporal branch was identified through direct stimulation [5]. In the SPKA, a small C- or W-shaped scalp incision at a site posterior to the facial nerve and craniotomy at ACF’s posterolateral part are performed, which provides lateral access to the ACF. Scalp incision location and craniotomy is determined for each case using a surgical navigation system. Based on the anatomy, the craniotomy is performed around the ICP and its cranial side (suprapterion keyhole, Fig. 1c and d).

Subsequently, scalp incision, craniotomy, tumor resection, and wound closure were performed using a 3D exoscope (ORBEYE; Olympus, Tokyo, Japan) (Fig. 1e). The tumor’s posterior and lateral part was removed as much as possible under the exoscopic view. After the tumor resection with the exoscope, the endoscope was inserted into the subfrontal space or frontal subdural space. The endoscope was fixed to a pneumatic articulated arm (Mitaka UniARM; Mitaka Kohki Co., Tokyo, Japan), enabling the surgeon to perform bimanual manipulation. The tumor remnants in the blind spots of the exoscope (medial and contralateral part) were removed using a rigid 0° or 30° endoscope (outer diameter, 4 mm; length, 18 cm; Karl Storz, Tuttlingen, Germany) (Fig. 1f). The endoscope provided the clear views on bilateral olfactory tracts and olfactory grooves; therefore, olfactory preservation was possible depending on the case.

The tumor removal procedures of three representative cases are shown below:

### Case 1: planum sphenoidale meningioma (Figs. 2 and 3*, *Video 1)

**Fig. 2:**
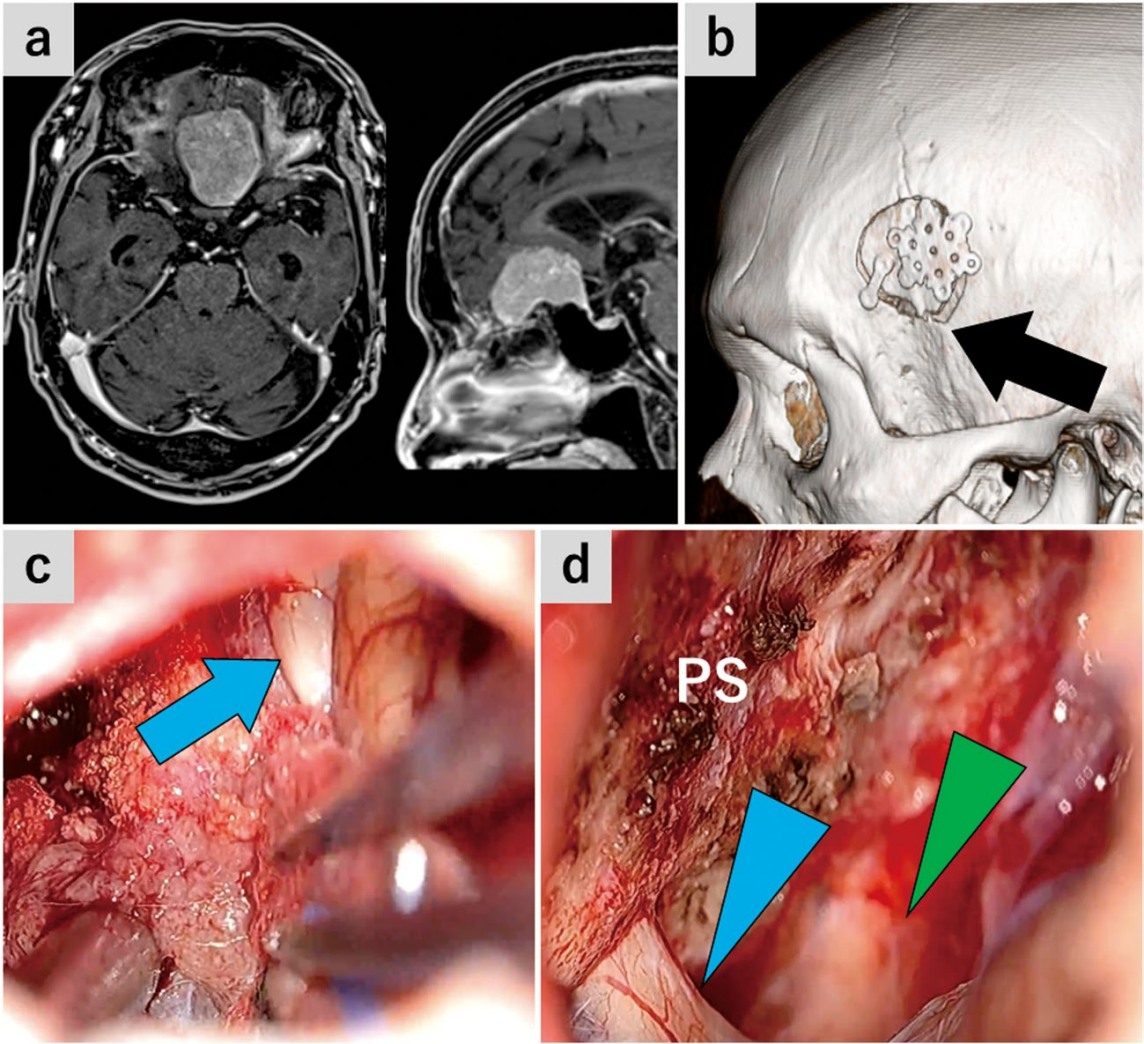
Case 1: Planum sphenoidale meningioma (**a**) Preoperative gadolinium-enhanced T1-weighted magnetic resonance image (MRI). **b** Postoperative 3D computed tomography shows craniotomy. Black arrow, ICP. (c and d) The tumor’s posterior part was exoscopically removed. **c** The posterior part of the tumor was separated from the bilateral olfactory tracts and frontal lobule. Blue arrows, left olfactory tract. (d) The posterior part of the tumor attaching to the planum sphenoidale was removed. Blue arrowhead, left optic nerve; green arrowhead, right optic nerve; PS, planum sphenoidale

**Fig. 3:**
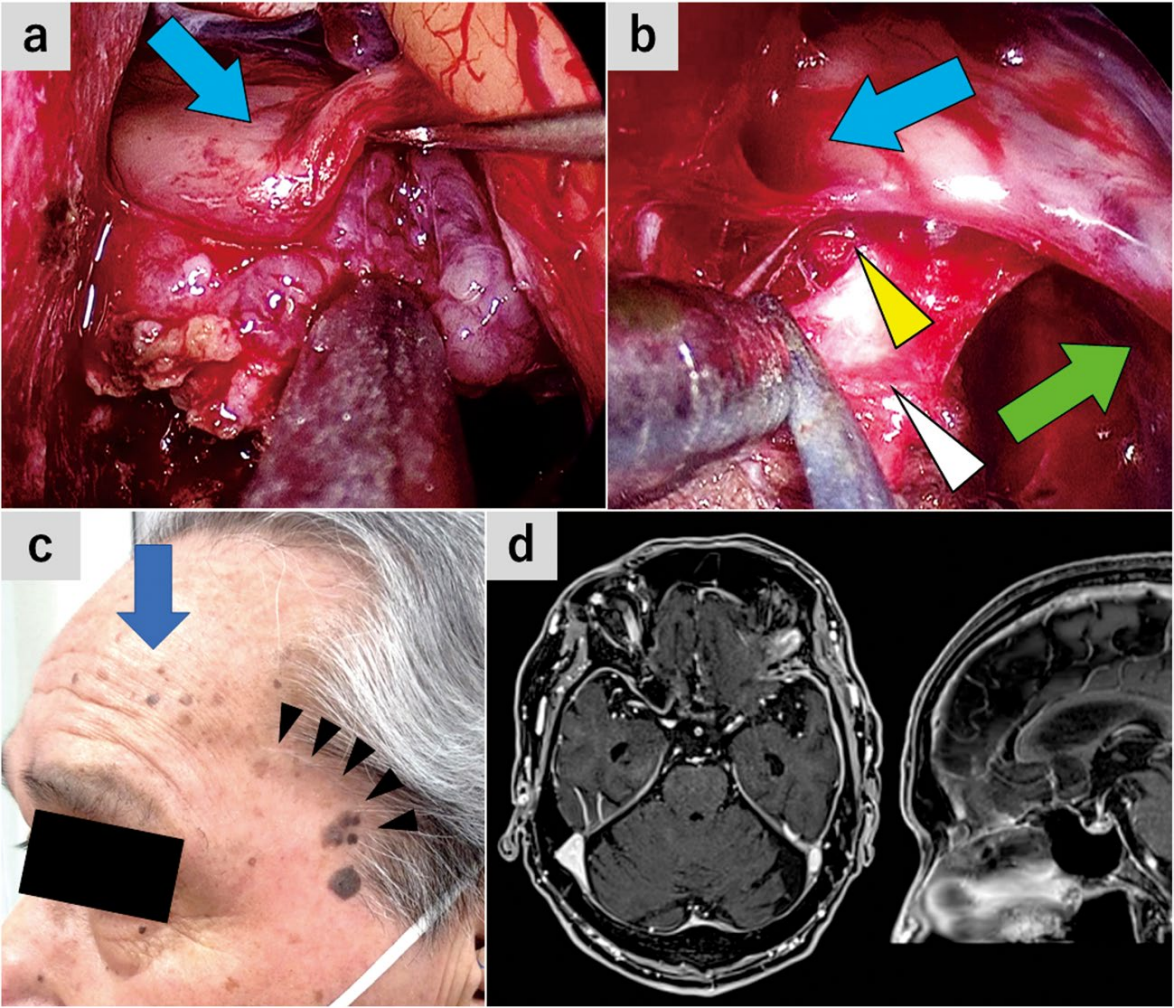
Case 1: Planum sphenoidale meningioma (**a** and **b**) The endoscope was inserted into the subfrontal space and remnant tumor was resected. **a** The posterior part of the tumor was separated from the bilateral olfactory tracts and frontal lobule. Blue arrow, left olfactory tract. **b** The tumor invading into the posterior edge of the left olfactory groove was removed. Blue arrow, left olfactory bulb; white arrowhead, posterior edge of the left olfactory groove; yellow arrowhead, posteroinferior edge of the falx. **c** 2 months postop, showing normal frontalis muscle contraction and a surgical scar. Blue arrow, Frontalis muscle contraction; black arrowheads, a surgical scar along the hair line. **d** Postoperative gadolinium-enhanced T1-weighted MRI

This was planum sphenoidale meningioma case accompanied by headache. Despite the tumor extension to the posterior edge of the bilateral olfactory grooves, the patient showed a normal olfactory function. The tumor was excised through SPKA, while preserving the bilateral olfactory nerves.

### Case 2: olfactory groove meningioma (Figs. 4 and 5*, *Video 2)

**Fig. 4:**
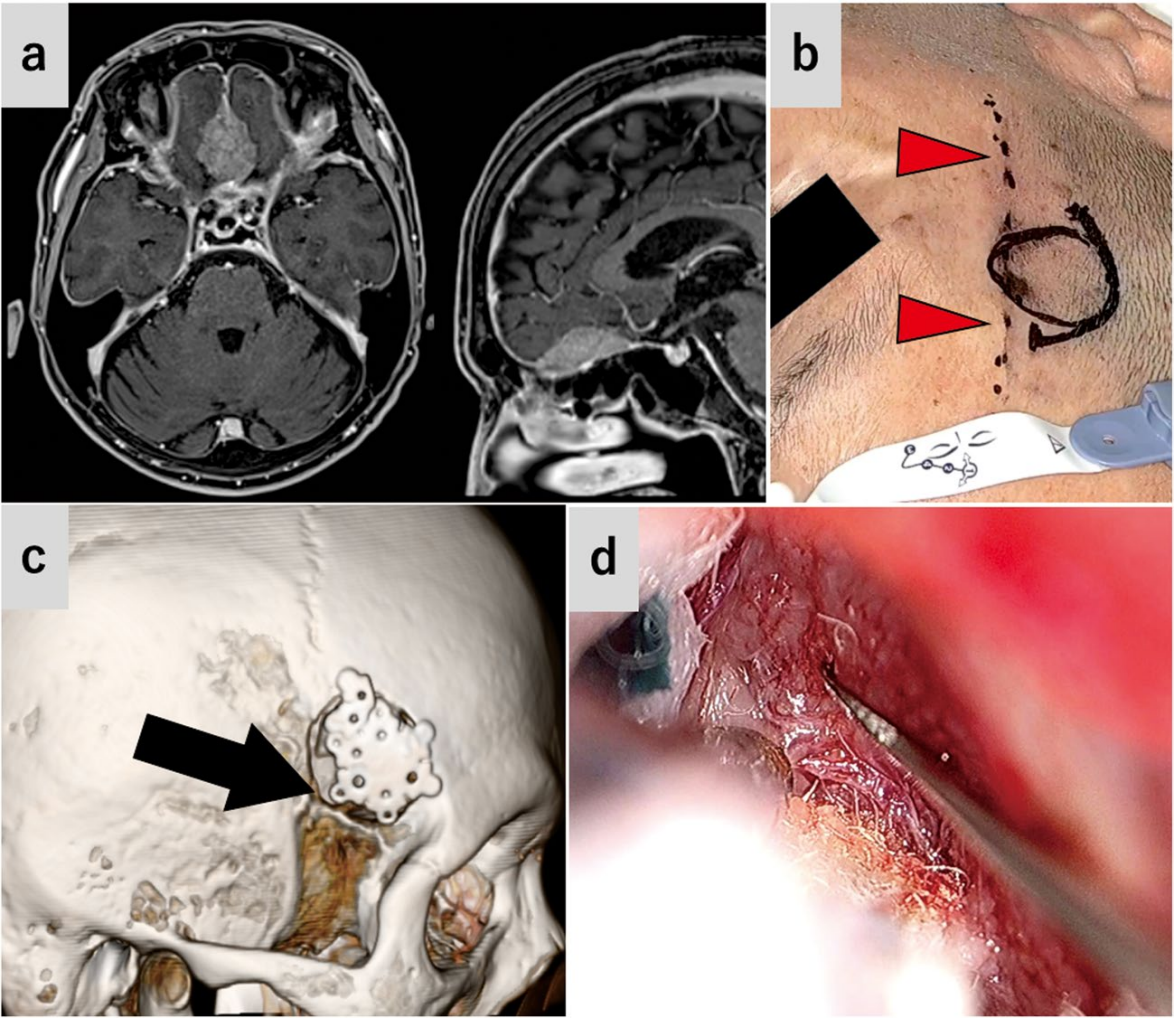
Case 2: Olfactory groove meningioma (**a**) Preoperative gadolinium-enhanced T1-weighted MRI. **b** Preoperative image. The solid line shows the designed skin incision and craniotomy size. Red arrowheads, the temporal branch of facial nerve marked by a dotted line. **c** Postoperative 3D computed tomography shows craniotomy. Black arrow, ICP. **d** The posterior part of the tumor attaching the planum sphenoidale was exoscopically removed

**Fig. 5:**
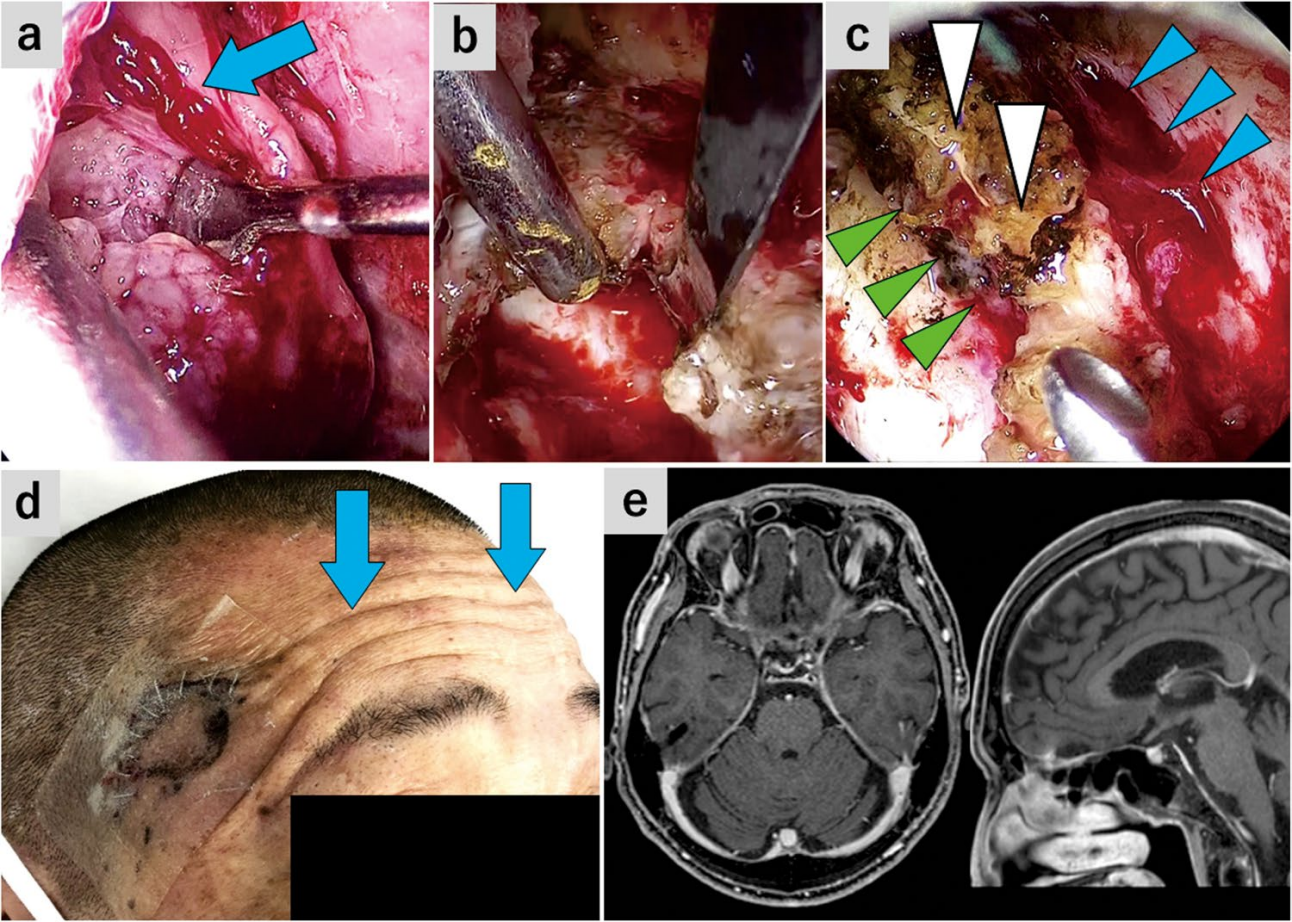
Case 2: Olfactory groove meningioma (**a**–**c**) The endoscope was inserted into the subfrontal space and the remnant tumor was resected. **a** The anterior part of the tumor involving the right olfactory tract was dissected. Blue arrow, right olfactory tract. **b** The posteroinferior edge of the falx is transected to remove the tumor invading the left olfactory groove. **c** The left side of the tumor was also excised. Blue arrowheads, right olfactory groove; white arrowheads, cut surface of the falx; green arrowheads, left olfactory groove. **d** The image immediately after surgery showing the wound location and normal frontalis muscle contraction. Blue arrows, Frontalis muscle contraction (**e**) Postoperative gadolinium-enhanced T1-weighted MRI

This was a case of olfactory groove meningioma accompanied by anosmia and secondary epilepsy. The tumor was removed without olfactory preservation through the SPKA.

### Case 3: frontal meningioma (Fig. 6*, *Video 3)

**Fig. 6:**
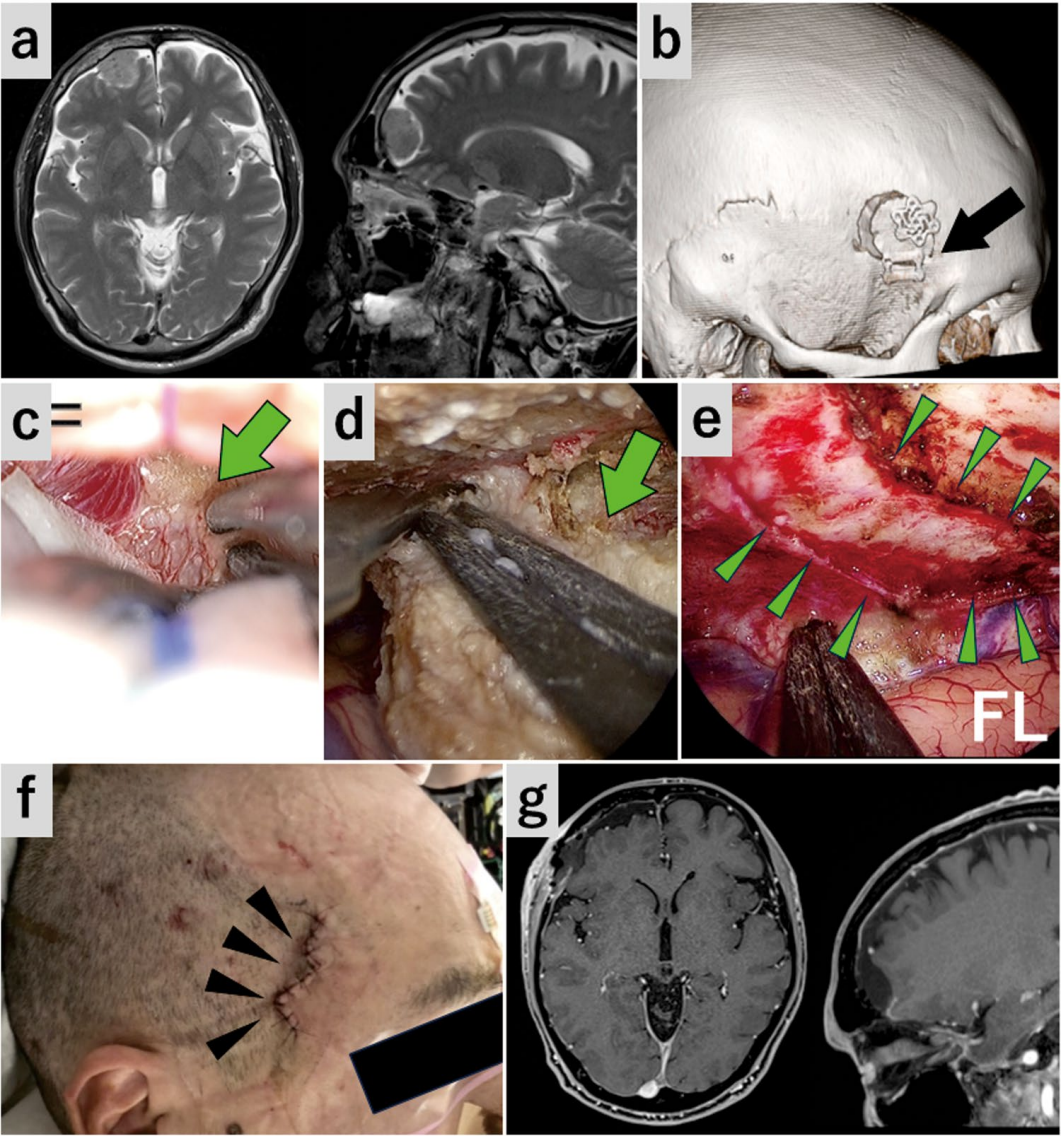
Case 3: Frontal meningioma (**a**) Preoperative T2-weighted MRI. **b** Postoperative 3D computed tomography shows craniotomy. Black arrow, ICP. **c** The lateral side of the tumor was excised under the exoscopic vision. Green arrow, tumor (**d** and **e**) The endoscope was inserted into the frontal subdural space. **d** The tumor was detached from the dura mater. Green arrow, tumor (**e**) The attachment dura mater was endoscopically resected. Duraplasty of the resected area was not necessary owing to the tight connection of the normal dura and the bone. Green arrowheads, cut margin of the dura mater; FL, frontal lobe. **f** Image immediately after surgery. Black arrowhead, W-shaped incision. **g** Postoperative gadolinium-enhanced T1-weighted MRI

This was a case of meningioma on the posterior wall of the frontal sinus. Due to the continuous tumor growth, SPKA was performed to resect the tumor.

In the SPKA, a large scalp incision or eyebrow incision is unnecessary, and the frontal sinus is rarely opened. The SPKA uses techniques and tools for a microscopic subfrontal approach, making it applicable to numerous neurosurgical institutions. While a microscope is a viable option instead of an exoscope, an endoscope for excising tumors around the olfactory grooves is preferable to an exoscope or a microscope. The following are the reasons why for using an exoscope: 1) the same surgical monitor is used for the exoscope and endoscope; therefore, switching as required between them is easy, 2) it allows for 3D image sharing, and 3) it is ergonomically superior, allowing the surgeon to view a wide area through a small craniotomy and to perform surgery in a comfortable posture [3, 4].

## Indications

The SPKA is used for ACF’s anteromedial part. The main indications are lesions in the bilateral planum sphenoidale or the olfactory grooves. Moreover, it is also applicable even for lesions located anterior to the olfactory groove as in Case 3.

## Limitations

Because the craniotomy size of the SPKA is ~ 2 cm, large (> 3 cm) and hard tumors are unsuitable. Easily bleeding tumors are also unsuitable; we evaluate the abundant vessels around the tumor in preoperative CTA as high bleeding risk. Although preoperative embolization and piecemeal resection can be applied to manage this problem in selected cases, we still lack the experience and knowledge to efficiently perform such procedures. Specifically, other approaches, including the interhemispheric approach, should be considered for larger lesions extending anterior to the bilateral cribriform plate. In the case of anterolateral lesions of ACF, supraorbital approach should be suitable.

## How to avoid complications

A preoperative careful assessment of the veins around the Sylvian fissure, cribriform plates, and lateral or inferior surfaces of the frontal lobe prevents inadvertent surgical bleeding and venous congestion. While contrast-enhanced computed tomography is sufficient, angiography is better. We aim for delicate manipulation to preserve the olfactory nerves. Furthermore, careful hemostasis and dural closure prevent postoperative bleeding and cerebrospinal fluid (CSF) leak.

## Specific information for the patient

There is a general surgical risk involved, such as infection and bleeding. Moreover, postoperative facial paralysis can occur due to the retraction of the facial nerve’s temporal branch, and postoperative olfactory dysfunction might occur due to the intraoperative damage of the olfactory pathway. In some patients, skin incisions might extend beyond their hairline and impair their cosmetic appearance.

## 10 key points summary

1. The SPKA is a less-invasive surgical method that is laterally applied to the ACF.
2. The main indication of the SPKA is lesions around the bilateral planum sphenoidale and olfactory groove.
3. The facial nerve’s temporal branch should be identified through direct stimulation to prevent frontalis paralysis.
4. From the posterior to the temporal branch of the facial nerve, a C- or W-shaped skin incision along the hairline was made.
5. A suprapterion keyhole (small craniotomy around the ICP) was performed.
6. The dura mater was cut to expose the frontal lobe.
7. The tumor’s posterolateral part was exoscopically dissected and resected.
8. The endoscope allows the removal of the tumor’s medial and contralateral part.
9. If necessary, a partial transection of the falx cerebri was performed to remove the tumor around the contralateral olfactory groove.
10. A layer-wise wound closure prevented CSF leak and preserved the patient’s cosmetic appearance.

## Supplementary information

Below is the link to the electronic supplementary material.

Supplementary file1 (MP4 80836 KB)

Supplementary file2 (MP4 73508 KB)

Supplementary file3 (MP4 92724 KB)

## Data Availability

The data supporting the findings of this study are available from the corresponding author, KI, upon reasonable request.

## Abbreviations

ACF: Anterior cranial fossa
SPKA: Suprapterional keyhole approach
ICP: Inferior coronal point
3D: Three-dimensional
CT: Computed tomography
MRI: Magnetic resonance imaging
CSF: Cerebrospinal fluid
